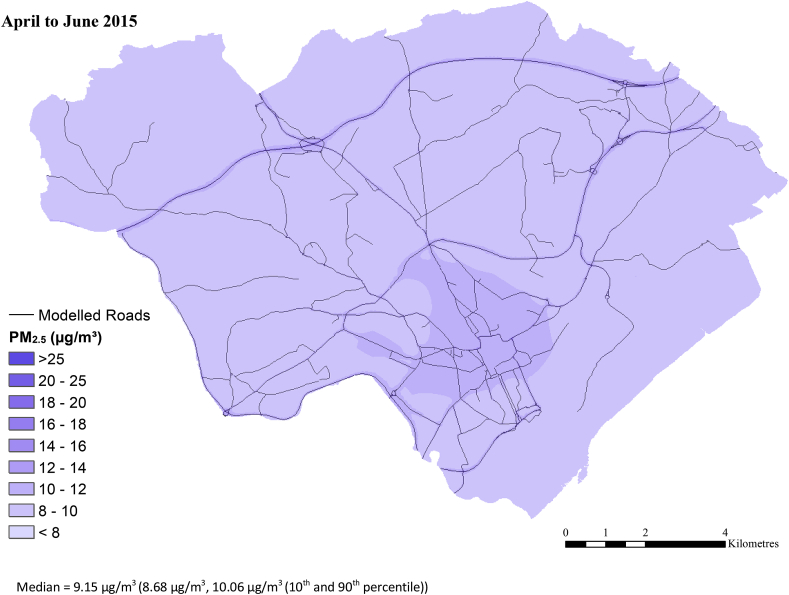# Impact of air pollution on educational attainment for respiratory health treated students: A cross sectional data linkage study

**DOI:** 10.1016/j.healthplace.2020.102355

**Published:** 2020-05

**Authors:** Amy Mizen, Jane Lyons, Ai Milojevic, Ruth Doherty, Paul Wilkinson, David Carruthers, Ashley Akbari, Iain Lake, Gwyneth A. Davies, Mohammad Al Sallakh, Richard Fry, Lorraine Dearden, Sarah E Rodgers

**Affiliations:** a Swansea University Medical School, Singleton Park, Swansea, UK; b Department of Social and Environmental Health Research, London School of Hygiene and Tropical Medicine, London, UK; c School of GeoSciences, The University of Edinburgh, Edinburgh, UK; d Cambridge Environmental Research Consultants, Cambridge, UK; e School of Environmental Sciences, University of East Anglia, Norwich, UK; f The Institute for Fiscal Studies, 7 Ridgmount Street, London, WC1E 7AE, UK; g Department of Public Health and Policy, University of Liverpool, Liverpool, UK

**Keywords:** Air pollution, Pollen, Data linkage, Educational attainment, Asthma, Seasonal allergic rhinitis

## Abstract

**Introduction:**

There is some evidence that exam results are worse when students are acutely exposed to air pollution. Studies investigating the association between air pollution and academic attainment have been constrained by small sample sizes.

**Methods:**

Cross sectional educational attainment data (2009–2015) from students aged 15–16 years in Cardiff, Wales were linked to primary health care data, modelled air pollution and measured pollen data, and analysed using multilevel linear regression models. Annual cohort, school and individual level confounders were adjusted for in single and multi-pollutant/pollen models. We stratified by treatment of asthma and/or Seasonal Allergic Rhinitis (SAR).

**Results:**

A unit (10μg/m^3^) increase of short-term exposure to NO_2_ was associated with 0.044 (95% CI: −0.079, −0.008) reduction of standardised Capped Point Score (CPS) after adjusting for individual and household risk factors for 18,241 students. This association remained statistically significant after controlling for other pollutants and pollen. There was no association of PM_2.5_, O_3_, or Pollen with standardised CPS remaining after adjustment. We found no evidence that treatment for asthma or SAR modified the observed NO_2_ effect on educational attainment.

**Conclusion:**

Our study showed that short-term exposure to traffic-related air pollution, specifically NO_2,_ was associated with detrimental educational attainment for students aged 15–16. Longitudinal investigations in different settings are required to confirm this possible impact and further work may uncover the long-term economic implications, and degree to which impacts are cumulative and permanent.

## 1 Introduction

The adverse effects of exposure to air pollution are a global public health concern in both developing and developed nations (Zhang et al., 2018). In the UK, the annual cost of the adverse effects of air pollution on human health is estimated to be about £15 billion (Department for Environment Food and Rural Affairs (DEFRA), 2010). Children and young people are particularly vulnerable to the effects of air pollution (Currie et al., 2013) because of its impact on physical and mental development which can cause health implications across the lifespan (Guerreiro et al., 2016). Detrimental impacts from exposure to air pollution have been reported for early life health outcomes such as pregnancy outcomes (Rogers and Dunlop, 2006), lung development and function (Portnov et al., 2009), the central nervous system (Block and Calderón-Garcidueñas, 2009), life expectancy at birth, and infant mortality (Fotourehchi, 2016). Furthermore, air pollution has also been reported to have adverse effects on mental development (Guxens and Sunyer, 2012), language development (Calderón-Garcidueñas et al., 2008), attention and sensory perception (Wang et al., 2009). Higher concentrations of NO_2_ and PM_2.5_ at the home location are thought to have both acute and irreversible effects on childhood cognition (Currie et al., 2014). These early life health outcomes are also related to long-term outcomes including; labour force participation, productivity (Hanna and Oliva, 2015), earnings (Currie et al., 2013) and poorer wellbeing and mental health.

Air pollution not only impacts on physical and mental development in childhood but also exacerbates respiratory conditions like asthma and Seasonal Allergic Rhinitis (SAR), more commonly referred to as hay fever. Hay fever is the most common chronic condition in children (Jáuregui et al., 2009) and is most prevalent among school students (Skoner, 2001). There is increasing evidence that air pollutants such as ozone (O_3_) may enhance the allergenicity of pollen, which in turn may impact cognitive development (Beck et al., 2013; Sturdy et al., 2012).

While we acknowledge there are mechanisms acting over the longer term causing air pollution to make permanent changes (Calderón-Garcidueñas et al., 2011), in this paper we focused on investigating the short-term effects of lower levels of air pollution. A plausible mechanism includes, interruption to learning/cognition due to asthma/hayfever related illness, resulting in a detrimental effect on educational outcomes. Air pollution has a negative impact on cognitive ability, including forgetfulness, inattentiveness and careless errors (Sunyer et al., 2017) which are important skills used in academic learning and examinations. Furthermore, exposure to air pollution has been negatively associated with school absences (Currie et al., 2009). In Israel, a longitudinal study of 71,383 students reported that high levels of PM_2.5_ were negatively associated with exam results (Lavy and Roth, 2014). Children with asthma (Ruijsbroek et al., 2015) and/or hay fever (Bensnes, 2016) have been found to have poorer educational outcomes. To our knowledge, the combined effect of the modification of air pollution on school performance by asthma or SAR, has not been investigated previously, possibly due to a lack of data linkage across these different domains.

In this study, the precision of the spatiotemporal data linkage is novel due to using high spatial resolution air pollution estimates modelled with a high degree of accuracy for specific periods, tailored to the timing of the outcome measures. We have linked air pollution estimates with temporally adjacent routinely collected health and education outcomes for each student. Routinely collected data are those that are collected for purposes other than research or without a specific research question (Benchimol et al., 2015). We used these data in a temporal investigation to explore whether acute exposure to air pollution had an impact on educational outcomes, and if children treated for Asthma/SAR were impacted to a greater or lesser extent. Previous studies have investigated the effect of air pollution on cognition by collecting data from participants enrolled in small studies (Freire et al., 2010); however, these studies have been restricted to small numbers of participants. Another study used repeated outcome measures from a birth cohort and linked these to a census tract averaged air pollution risk measure at a single time point (Grineski et al., 2020). Our study used a city-wide cohort of all students who sat General Certificate of Secondary Education (GCSE) examinations as a proxy for cognition. These examinations are taken by most students at the end of compulsory schooling in England, Wales, and Northern Ireland.

Our overarching aim was to determine the acute impact of air pollution on educational attainment by investigating how the relationship between short-term exposure to air pollution and GCSE results varied over time. CORTEX was a repeated cross-sectional retrospective data linkage study with two principal research questions:

1. Is exposure to air pollution in the term prior to GCSE examinations negatively associated with exam performance?
2. For children treated for SAR and/or Asthma, is exposure to air pollution in the term prior to GCSE examinations negatively associated with exam performance?

## 2 Methods

We used routinely collected data held in the SAIL Databank to answer our research questions. We calculated multiple location, daily exposures to air pollution for each student. We linked these exposures to students being actively treated for asthma or SAR, and their educational attainment score. A detailed methodology was previously documented (Mizen et al., 2018).

### 2.1 Study area

Cardiff has a population of 346,100 and is located on the south coast of Wales, in a maritime temperate climate with a prevailing south westerly wind. There is some industry, including steel manufacturing, but it is not a particularly polluted city. Current WHO guidelines set to protect the public from health effects of pollution stipulate that the annual city mean should not exceed 40 μg/m^3^ for NO_2_ (World Health Organization, 2018). Cardiff is representative of a number of small cities throughout the world whose populations experience comparatively lower level exposure to pollutants.

We used the Secure Anonymised Information Linkage (SAIL) Databank to retrieve education data for all students in Key Stage Four (15–16 years of age) who attended state secondary schools and linked them with their environmental and primary health care data. Our inclusion criteria included students in Key Stage Four who: studied in a state school in Cardiff (23 schools); sat the General Certificate of Secondary Education (GCSE) examinations during 2009–2015; lived in Cardiff; and were registered with a General Practitioner (GP, primary care physician) in Wales during the same period.

The SAIL Databank contains anonymised longitudinal, routinely collected, health, social, environmental, and education data on the Welsh population (Lyons et al., 2009). This infrastructure enabled us to bring together individual-level pollution and pollen exposures, and health and educational outcomes for six years. SAIL also contains the Welsh Demographics Service (WDS) dataset, a population register, which includes home address histories provided by patients registered with a GP in Wales, a service that is free at the point of care in the UK. Using the home address recorded in the WDS and school information in the National Pupil Dataset (NPD), we were able to link air pollution and pollen data at both home and school locations to tailor exposures for individual students.

### 2.2 Educational attainment

We obtained a continuous measure of educational attainment, derived from GCSE examination scores from the National Pupil Database (NPD) for Wales (2009–2015). Each student took their exams during May and June at age 15–16 years in their final year of mandatory schooling in the UK; although the exact date of the examination period varied between years. Their GCSE examination scores were transformed into a Capped Points Plus score (CPS), a continuous measure of attainment which is derived from a student's best eight subjects including Mathematics, and English Language or Welsh as a first language. We standardised the CPS using the available mean within each exam-year cohort to create z-scores. Thereby removing discrepancies due to differences in examination papers in different years.

### 2.3 Air pollution and pollen (aeroallergen) exposure

**Fig. 1:**
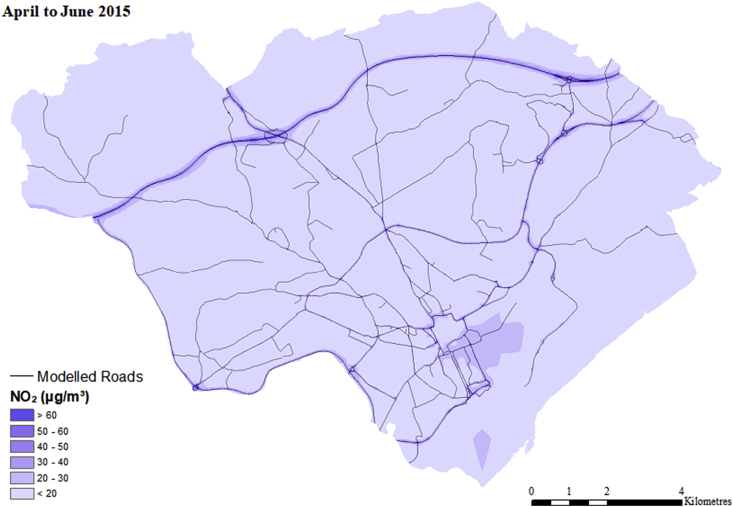
Spatial distribution of the annual average NO_2_ concentration for Cardiff, UK (2015). Summary statistics for 2015: 13.23µg/m^3^, 10.96 µg/m^3^, 18.74 µg/m^3^ (Median, 10^th^ and 90^th^ percentiles). All pollutants were estimated at a 20m spatial resolution. Yearly summary statistics are in the Appendix.

Hourly ambient concentration of fine particulate matters that have a diameter of less than 2.5 μm (PM_2.5_), nitrogen dioxide (NO_2_), and ozone (O_3_) were modelled by the Atmospheric Dispersion Modelling System (ADMS) Urban model (Carruthers et al., 1994) in 20 m spatial resolution for Cardiff city during the study period (2009–2015). Fig. 1 shows the spatial variation of modelled NO_2_ levels across Cardiff for 2015. Appendix 1 documents spatial variation across Cardiff for modelled values of NO_2_, PM_2.5_ and O_3_ from 2009 to 2015. An indication of model accuracy is provided by comparison of modelled annual average NO_2_ concentrations with measurements over a five-year period from around 50 diffusion tubes located across Cardiff. For each year these typically showed a fractional bias over all sites of about 0.05 corresponding to an underestimation of less than 2 μg/m^3^. Comparisons with measurements from one automatic reference monitor on Cardiff for NO_2_ and PM showed a similar level of performance. We would expect the model predictions for O_3_ to be similarly accurate given the model predictions of O_3_ are based on measured rural values and the variations in O_3_ across cities are strongly anti-correlated with NO_2_ concentrations.

**Fig. 2:**
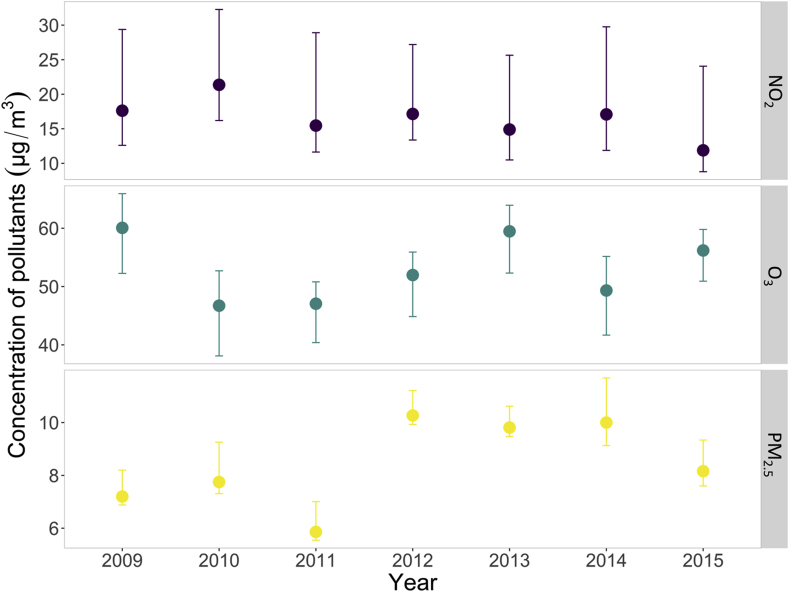
Min, Max and Mean of annual personal exposure concentration for study students (n=18,241) from 2009-2015. Exposure is based on air pollution estimates weighted by time spent at home, and school from 9am-3pm on weekdays in term time.

Hourly pollution levels at the home and school location were combined to create a daily exposure profile for each student for PM_2.5_, NO_2_ and O_3_ (Rodgers et al., 2009). We assumed the student was at school during school time (9am-3pm on weekdays in school term); and at home during off-school time (3pm-9am on weekdays in school term and whole day for weekend, bank holidays and school holidays). Exposure was characterised by a weighted average for the pre-examination period (from the start of the summer term, generally 1st April, to the day before their first exam) and for the duration of their examination period (first exam to last exam) (Mizen et al., 2018). Fig. 2 highlights temporal variation of the weighted averages throughout the study period.

Time series data for a single pollen measuring site in Cardiff was available from the European Allergy Network for 2006–2010 (“EAN - European Aeroallergen Network - Home,” 2019) and from the UK Met Office for 2011–2015 (“Weather and climate change - Met Office,” 2019). Exposure to pollen was characterised by total pollen counts for all pollen sources combined (tree, grass and weed) for each of the pre-examination and examination time periods.

### 2.4 Other risk factors

The definition of an asthma patient varies greatly in the literature [42]. We selected students who were ever diagnosed with or treated for asthma or SAR in the year before the summer examination period. The examination period was defined using the Welsh Joint Education Committee (WJEC) June series exam board timetable. Case definitions for asthma were based on those used in the Wales Asthma Observatory (Al Sallakh, 2018). Asthma and SAR diagnosis and treatment were queried from the Welsh Longitudinal General Practice (WLGP) dataset in the SAIL Databank. Students who were treated for asthma or SAR were flagged from Read codes in the primary care dataset (Mizen et al., 2018).

We considered small-area level and household level measures of deprivation as potential confounders. At the small area level (Lower Super Output Area, LSOA ~1500 people) we used the Welsh Index of Multiple Deprivation (WIMD). Eligibility for Free School Meals (FSM) was a household level measure of deprivation, recorded as a part of the NPD. We also included Special Educational Needs (SEN) as recorded in the NPD, and sex, recorded in the WDS, as confounders. The SEN is a derived binary variable indicating whether the student is recorded as having a condition that could affect their ability to learn.

### 2.5 Statistical analysis

We applied multilevel linear regression analyses to assess the impact of selected pollutants/pollen level on educational attainment, accounting for similarities among students who went to the same school and were in the same exam year. We used single pollutant/pollen models to assess whether temporal associations between each pollutant/pollen and educational attainment were observable, before adjusting for major individual and household risk factors. Where we detected associations, we applied multipollutant models to adjust for other pollutant(s)/pollen. We stratified the analyses by treatment for asthma and SAR to assess effect modification by health condition.

We accounted for clustering within households at the cohort level because siblings (except multiple births) appeared in different cohorts. Twins clustered within a yearly cohort, however the number of twins within each cohort was small (<3% of the study population) and we decided it was unnecessary to account for clustering within households within an exam-year cohort. We conducted two separate sensitivity analyses. First, we combined three pollutant/pollen models to adjust for correlated pollutants/pollen. Second, we changed our assumption of student location from school to home for the 2 h immediately after the end of the school day (3–5pm). This helped us to assess the effect on exposure of assuming that students remained at school for clubs after hours.

We conducted all analyses in R version 3.5.0 within the privacy protecting secure SAIL Databank environment. Our study was approved by an independent Information Governance Review Panel (IGRP) application (approval number 0561).

## 3 Results

We analysed 18,241 students at age 15–16 years old who lived and studied in Cardiff and undertook their GCSE examinations between 2009 and 2015. Students were an average of 15.71 years old (SD 0.45). For each yearly exam cohort, the final z-score transformation of CPS had a mean of zero and a standard deviation of one. Across the seven cohorts, the CPS ranged from −3.7 to 2.2. Among the study population, 1505 students (8%) were treated for asthma and 2725 (15%) were treated for SAR (Table 1). Almost a fifth of the population were treated for either Asthma or SAR (n = 3595); 80% were treated for neither condition (n = 14,645); and 3% were treated for both conditions (n = 634). There were 3616 students (20%) with Special Educational Needs, and 2860 students (16%) were eligible for Free School Meals. Greater proportions of students were distributed in the least and most deprived quintiles (WIMD 1, 30%; WIMD 5, 38%) compared to the whole of Wales. These characteristics did not vary considerably between exam years.

**Table 1:** Characteristics of the study population (n = 18,241).

Demographics	n	%	
**Male**	9337	51%	
**Female**	8904	49%	
**Treated for Asthma**	1505	8%	
**Treated for SAR**	2725	15%	
**Treated for both Asthma and SAR**	634	3%	
**Treated for neither Asthma or SAR**	14,645	80%	
**Treated for either Asthma or SAR**	3596	20%	
**Eligible for Free School Meals**	2860	16%	
**Special Educational Needs**	3616	20%	
**WIMD 1 (most deprived)**	5454	30%	
**WIMD 2**	2181	12%	
**WIMD 3**	1549	8%	
**WIMD 4**	2142	12%	
**WIMD 5 (least deprived)**	6915	38%	

SAR, Seasonal Allergic Rhinitis; WIMD, Welsh Index of Multiple Deprivation.

**Fig. 3:**
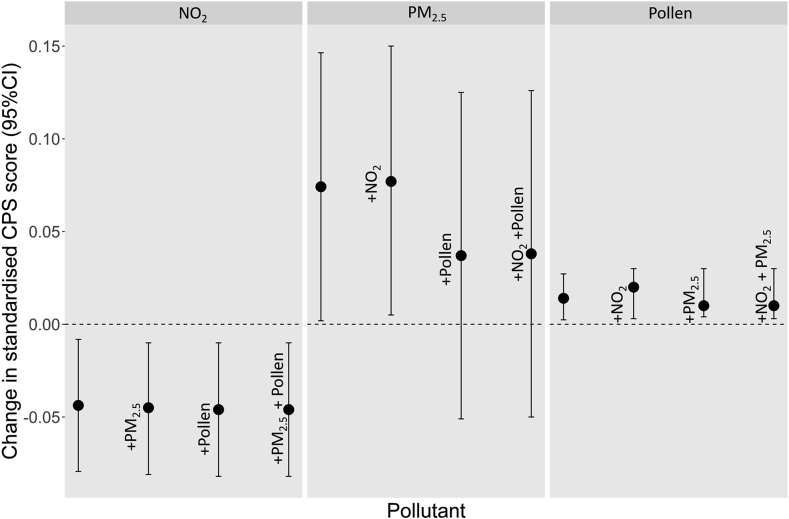
Change in standardised CPS score (95% CI) per 10mg/m3 before and after adjustment for other pollutants and/or pollen. All models were adjusted for potential confounders: socioeconomic status (WIMD), Free school meal eligibility, special education needs, and sex.

In our primary analyses of single pollutants, a unit (10μg/m3) increase of NO_2_ was associated with a 0.044 (95% CI: −0.079, −0.008%) reduction of standardised CPS score after adjusting for sex, household and neighbourhood deprivation, and special educational needs. We found a unit increase of PM_2.5_ (0.074) and pollen (0.015) were associated with a higher standardised CPS score (0.074, 95%CI: 0.002, 0.146; and 0.015, 95% CI: 0.002, 0.027) (Table 2). The association between NO_2_ and standardised CPS score remained after adjustment for PM_2.5_ and pollen in the multipollutant model. The positive impact of PM_2.5_ and pollen on standardised CPS score did not remain having adjusted for PM_2.5_ or pollen (Fig. 3).

**Table 2:** Change (95% CI) in educational attainment (standardised GCSE CPS) associated with a unit increase in air pollution and pollen exposure and other risk factors for students at age 15–16 years in Cardiff 2009–2015, single pollutant/pollen model (n = 18,241).

	NO_2_ model	PM_2.5_ model	O_3_ model	Pollen model
**NO**_**2**_**, per 10 μg/m**^**3**^	−0.044 (−0.079, −0.008)	–	–	–
**PM**_**2.5**_**, per 10 μg/m**^**3**^	–	0.074 (0.002, 0.146)	–	–
**O**_**3**_**, per 10 μg/m**^**3**^	–	–	0.004 (−0.017, 0.024)	–
**Pollen, 10**^**−5**^**grains/m**^**3**^	–	–	–	0.015 (0.002, 0.027)
**Female (vs Male)**	0.096 (0.073, 0.118)	0.096 (0.074, 0.119)	0.096 (0.073, 0.118)	0.096 (0.074, 0.119)
**Eligibility for Free School Meal**	−0.360 (−0.393, −0.326)	−0.360 (−0.393, −0.326)	−0.360 (−0.393, −0.326)	−0.360 (−0.393, −0.326)
**Special Education Needs (Yes vs No)**	−1.000 (−1.029, −0.971)	−1.000 (−1.029, −0.970)	−0.999 (−1.028, −0.969)	−1.000 (−1.029, −0.970)
**Free School Meals at school**	−0.001 (−0.001, 0.001)	−0.001 (−0.001, 0.001)	−0.001 (−0.001, 0.000)	−0.001 (−0.001, 0.001)
**Neighbourhood deprivation Q2**^a^	0.095 (0.055, 0.134)	0.093 (0.054, 0.133)	0.094 (0.054, 0.134)	0.093 (0.053, 0.133)
**Neighbourhood deprivation Q3**^a^	0.205 (0.160, 0.251)	0.202 (0.156, 0.248)	0.203 (0.157, 0.248)	0.202 (0.156, 0.248)
**Neighbourhood deprivation Q4**^a^	0.224 (0.181, 0.267)	0.223 (0.180, 0.266)	0.223 (0.180, 0.266)	0.223 (0.180, 0.266)
**Neighbourhood deprivation Q5**^a^	0.395 (0.358, 0.433)	0.395 (0.358, 0.432)	0.395 (0.357, 0.432)	0.395 (0.357, 0.432)

a Against neighbourhood deprivation quintile group 1, Q1 (the most deprived group). Quintile 5 (Q5) is the least deprived group.

Stratification by treatment suggested NO_2_ impacts were statistically significant only for students without asthma or SAR treatment. The positive associations with the standardised CPS score (i.e. high exposure to pollen was associated with higher exam score) observed in the single model, among students without asthma or SAR treatment, did not remain when additionally adjusted for NO_2_ or PM_2.5_ (Table 3).

**Table 3:** Change (95%CI) in educational attainment (standardised GCSE CPS) associated with unit increase^a^ in air pollution and pollen exposure by asthma and SAR treatment status.

	Treatment for asthma		Treatment for SAR	
	Yes	No	Yes	No
**NO**_**2**_	−0.040 (−0.161, 0.081)	−0.041 (−0.078, −0.004)	−0.015 (−0.100, 0.071)	−0.048 (−0.087, −0.009)
**NO**_**2**_**adjusted for PM**_**2.5**_	−0.043 (−0.163, 0.078)	−0.043 (−0.080, −0.005)	−0.015 (−0.101, 0.070)	−0.049 (−0.088, −0.010)
**NO**_**2**_**adjusted for pollen**	−0.042 (−0.163, 0.080)	−0.044 (−0.081, −0.006)	−0.016 (0.101, 0.071)	−0.050 (−0.089, −0.010)
**NO**_**2**_**adjusted for PM**_**2.5**_**and pollen**	−0.042 (−0.163, 0.078)	−0.044 (−0.081, −0.007)	−0.015 (−0.010, 0.007)	−0.050 (−0.089, −0.011)
**PM**_**2.5**_	0.189 (−0.062, 0.439)	0.067 (−0.009, 0.141)	0.143 (−0.040, 0.325)	0.064 (−0.015, 0.142)
**PM**_**2.5**_**adjusted for NO**_**2**_	0.191 (−0.060, 0.441)	0.070 (−0.006, 0.145)	0.143 (−0.039, 0.326)	0.067 (−0.011, 0.146)
**PM**_**2.5**_**adjusted for Pollen**	0.210 (−0.094, 0.511)	0.020 (−0.071, 0.112)	0.183 (−0.042, 0.407)	0.013 (−0.082, 0.108)
**PM**_**2.5**_**adjusted for NO**_**2**_**and pollen**	0.210 (−0.093, 0.512)	0.022 (−0.070, 0.113)	0.182 (−0.042, 0.406)	0.015 (−0.081, 0.110)
**O**_**3**_	−0.007 (−0.078, 0.064)	0.004 (−0.017,0.026)	−0.031 (−0.082,0.021)	0.010 (−0.012, 0.033)
**O**_**3**_**adjusted or NO**_**2**_	−0.025 (−0.107, 0.057)	−0.009 (−0.034, 0.015)	−0.048 (−0.108, 0.012)	−0.004 (−0.030, 0.021)
**O**_**3**_**adjusted for PM**_**2.5**_	−0.015 (−0.087, 0.057)	0.001 (−0.021, 0.023)	−0.039 (−0.091, 0.013)	0.007 (−0.016, 0.030)
**O**_**3**_**adjusted for Pollen**	−0.012 (−0.085, 0.061)	−0.002 (−0.024, 0.021)	−0.034 (−0.087, 0.018)	0.004 (−0.019, 0.027)
**Pollen**	0.01 (−0.03, 0.06)	0.016 (0.003,0.029)	0.0006 (−0.003,0.004)	0.002 (0.0003,0.003)
**Pollen adjusted for NO**_**2**_	0.001 (−0.003, 0.006)	0.002 (0.0004, 0.003)	0.0007 (−0.003, 0.004)	0.002 (−0.0004, 0.003)
**Pollen adjusted for PM**_**2.5**_	−0.0006 (−0.006, 0.005)	0.001 (−0.0002, 0.003)	−0.0012 (−0.005, 0.003)	0.002 (−0.0001, 0.003)
**Pollen adjusted for O**_**3**_	0.002 (−0.003, 0.006)	0.002 (0.0003, 0.003)	0.0011 (−0.002, 0.004)	0.002 (0.0002, 0.003)

a Per 10 μg/m^3^ for NO_2_, PM_2.5_ and O_3_, 1000 grains/m^3^ for Pollen.

We initially assumed the student would be at home 3–5pm. We observed little difference in the beta coefficients and confidence intervals when we re-ran our single pollutant models assuming the student remained at school (for after school clubs) from 3 to 5pm (data not shown).

## 4 Discussion

### 4.1 Main findings

Our study suggests that short-term exposure to NO_2_ levels that are below current policy thresholds (World Health Organization, 2018) have a detrimental impact on educational attainment at the age 15–16 years. Specifically, we found a 0.044 reduction of standardised CPS score per 10ug/m^3^ increase of NO_2_ exposure level. Although this reduction is modest, Cardiff is a moderate sized city (population 300,000) and is not considered to be particularly polluted compared to other cities of its size. This relationship was not found for the other pollutants (PM_2.5_ and O_3_) or pollen. We report a significant, positive association between exposure to pollen and CPS score. With respect to the impact of exposure to pollen on educational attainment for children treated for SAR and/or asthma, we found exposure to air pollution and/or pollen to have no effect on exam performance.

### 4.2 Comparison with other studies

Our results suggest that short-term exposure to NO_2_ has a negative impact on examination scores. This supports findings where long-term exposure to traffic related air pollution (including NO_2_) during development had detrimental effects on cognitive development (Sunyer et al., 2015). We report no significant association between PM_2.5_ and examination scores. This is in contrast to a panel study estimating the short-term effect of air pollution exposure on standardised test scores among Israeli high school examinations that found PM_2.5_ to be negatively associated with educational attainment (Lavy and Roth, 2014) with a larger negative impact on groups with higher asthma rates. We found no association between short-term exposure to O_3_ and educational attainment, although evidence suggests that long term exposure to O_3_ is damaging for cognitive development (Chen and Schwartz, 2009).

Our results suggest that educational outcomes for children not treated for SAR and/or asthma are positively associated with exposure to pollen. However, the effect size of this association is minute. With respect to the impact of exposure to pollen on educational attainment for children treated for SAR and/or asthma, our results echo studies that have reported SAR having no effect on cognition in adolescents, including exam performance (Blaiss et al., 2018). Another study that reported a negative association of SAR with school performance (Kim et al., 2017) used self-reported data and may have captured more cases of SAR than our study. In our study, it may be that only the more severe cases of SAR are recorded in the primary care data because over-the-counter treatments are available. On the other hand, free prescriptions in Wales mean that all children are eligible for free treatment for SAR from their doctor.

### 4.3 Strengths and limitations

This is the first study, to our best knowledge, to investigate air pollution impact on educational attainment using routinely collected data source with high spatial resolution air pollution exposure data tailored to the temporal focus of this study. We investigated associations between short-term exposure to ambient air pollution and educational attainment, as a proxy for cognition. We linked routinely collected educational and health data for 18,241 students aged 15–16 years in Cardiff with air pollution data modelled at 20m resolution aggregated into precise revision and examination periods. We included exposure to air pollution at home and school location for all students.

A strength of this study is the number of students (18,241) in the retrospective cohorts for whom we linked modelled pollution data at both their home and school locations. We modelled exposure at home and school, reflecting not just the pollutants but combining these into a dual location daily exposure using the high spatial resolution ADMS model. Furthermore, we linked these environmental exposures with individual health data to explore the impact of health conditions. This study has shown it is possible to link multiple location air pollution estimates, transform them into dual location exposures, and then link to individual-level health outcomes in an anonymised data safe haven to contribute to filling an important evidence gap.

A limitation of the study was that exposures were estimated by modelling, rather than continuous personal exposure monitoring. However, this was justified by the size of our study. Population based cohort studies need to use modelling because personal, measured exposures are not feasible at scale. Another limitation was that, unlike the air pollution data, the pollen data were not spatially or temporally resolved. Pollen modelling is less advanced in comparison to air pollution modelling (Desjardins et al., 2019). Furthermore, we did not consider the indoor environment (Sunyer et al., 2015), such as the smoking status of the students or their cohabitants.

Outcomes were measured by CPS. English and Maths are integrated into the score and we were not be able to specify which other subjects were examined. Furthermore, our results do not necessarily support the assertion that air pollution impacts on cognition, because it is not clear how CPS is associated with cognition measured by standard instruments. However, poorer examination results have implications for future education and economic work life benefits (Bensnes, 2016). It was not possible to adjust for curriculum changes, such as changes to the proportional contribution of coursework grades to final subject grades, or temporal changes associated with coursework deadlines. Similarly, it was not possible to identify if students had taken some exams early such as during the November or January examination series. We recommend education results are available on a per subject basis, to allow the examination of the effects of air pollution on specific learning outcome areas in future.

We did not model pollution along a student's route to school, or at locations where they may have spent their evenings other than at home. We assumed that each student spent their time at only two locations, but, in combination with temporal tailoring, this is likely to have reduced some errors (Park and Kwan, 2017). The high spatial and temporal resolution of the data used in this study has allowed us to more accurately represent the spatial and temporal patterns of pollution exposure variation to students and their outcome data. We did not include noise pollution data or a proxy thereof. Noise data for Wales are currently modelled using traffic, railway and industrial area data for select regions of the study area at ad-hoc timepoints (Review of strategic noise maps in Wales, 2015). The lack of consistent temporal or spatial coverage of these data meant we were not able to control for the effects of noise pollution from the perspective of the household/individual unit of analysis as used in our study.

A limitation of the routinely collected clinical data is that only those with a GP diagnosis or prescription will be captured. Allergic rhinitis may be self-diagnosed in up to 50% of the population, and over-the-counter medications are commonly used, although this is mitigated by the availability of free prescriptions in Wales since April 1, 2007. However, those with more severe disease, and higher susceptibility to pollen and pollution, are more likely to be identified.

It is possible that taking over-the-counter antihistamines may have influenced examination outcomes; however, it was not possible to account for that in this study. In addition, dust mites are a common trigger of year-round allergies and asthma, but data about these were not available. Also, students may have been suffering from additional cardio-respiratory conditions or other comorbidities, which were not included in this analysis and could have had an effect on asthma or SAR symptoms and severity or educational attainment.

### 4.4 Implications

This research suggests that air pollution experienced in the short-term is having a detrimental effect on school student's educational outcomes. It is crucial this is addressed through evidence-based policies and interventions. Consideration needs to be given to innovative ways to reduce the exposure of children to air pollution and ultimately investment needs to be made to change the prevailing cultural and industrial legacies that contribute to high emissions of pollutants. This is particularly important considering that the short-term impacts culminate into longer term exposure and permanent changes even within cities with low to moderate pollution (Sunyer et al., 2015), as well as those with very poor air quality (Calderón-Garcidueñas et al., 2011).

Challenges remain in linking combinations of national and local air quality improvement initiatives to air pollution reductions, however, here we have shown we can integrate socio-demographic, health and environment data to better understand the relationship between lower level exposures to pollutants (Chief Medical Officer, 2017). Our results highlight that the degree of residential area deprivation has a major and consistent impact on educational attainment across all models. Current UK policies to reduce air pollution are focused on economic and overall health improvement (DEFRA, 2020). Our results suggest that future analyses should be based on deprivation levels to improve our insights into the effects of air pollution. This would provide evidence to inform policies that improve air quality for the most deprived populations to reduce health inequalities (Chief Medical Officer, 2017). The postponement of the introduction of the Clean Air Zones in the UK due to the COVID-19 pandemic (The Guardian, 2020) could be an opportunity to include a health inequality assessment in future policy (Whitehead, 2014).

### 4.5 Future research

Future research should consider exploring urban *and* rural exposures to pollution and it has been noted that O_3_ concentrations are often lower in urban areas because it is depleted by NOx. It may be that different pollutants have different effects depending on their spatial distribution. The effect of pollution on cognition by sex should be explored as males may be more affected by air pollution (Chen et al., 2017).

To complete a robust longitudinal analysis, it would be necessary to expand the geographic area of study to maintain sufficient student numbers at each key stage. Alternatively, because are no equivalent cognition outcome data for the whole population at earlier ages, we instead recommend developmental and cognition outcome measures are collected from a representative sample using survey instruments following air pollution modelling (Gulliver et al., 2018). High spatial and temporal modelled pollution data are not available routinely for all urban areas, however, we anticipate that these results will prove to be valuable, thereby encouraging additional urban area modelling. We recommend future work include data on emergency hospital admissions and attendances for respiratory conditions, which occurred too infrequently in the current single city study sample. These outcomes are not subject to treatment seeking behaviour. Utilising secondary healthcare data can be used as a measure of condition severity but also highlight temporal flares of conditions and associated temporal changes in air pollutant concentrations. Ultimately, current traffic reductions from the COVID-19 social isolation measures are likely to have improved air quality in many cities, although simultaneous disruptions to education means using attainment as an outcome is unfeasible.

## 5 Conclusion

Short-term exposure to NO_2_, coincident with revision periods, was negatively associated with educational attainment. We did not find associations with PM_2.5,_ O_3_, or pollen. We found no evidence that treatment for asthma or SAR modified the observed NO_2_ effect on educational attainment. Further longitudinal investigations are required to understand the complicated relationship between air pollution, pollen, asthma, SAR, and educational attainment.

## Ethics & availability of data

This study makes use of anonymised data held in the Secure Anonymised Information Linkage (SAIL) Databank. We would like to acknowledge all the data providers who make anonymised data available for research. The data used in this study are available in the SAIL Databank at Swansea University, Swansea, UK. All proposals to use SAIL data are subject to review by an independent Information Governance Review Panel (IGRP). Before any data can be accessed, approval must be given by the IGRP. The IGRP gives careful consideration to each project to ensure proper and appropriate use of SAIL data. When access has been approved, it is gained through a privacy-protecting safe haven and remote access system referred to as the SAIL Gateway. SAIL has established an application process to be followed by anyone who would like to access data via SAIL https://www.saildatabank.com/application-process.

## Author contribution

AM was responsible for writing the paper. All the co-authors made substantial contributions to writing the methods section and editing the whole paper. All authors then read the final version and approved it for submission and publication.

## Funding

This study was funded by 10.13039/501100000589Chief Scientist Office of Scotland, the 10.13039/501100000265Medical Research Council and the 10.13039/501100000270Natural Environment Research Council (R8/H12/83/NE/P010660/1).

We acknowledge the support from The Farr Institute @ CIPHER: MR/K006525/1, which is supported by a 10-funder consortium: 10.13039/501100000341Arthritis Research UK, the 10.13039/501100000274British Heart Foundation, 10.13039/501100000289Cancer Research UK, the 10.13039/501100000269Economic and Social Research Council, the 10.13039/501100000266Engineering and Physical Sciences Research Council, the 10.13039/501100000265Medical Research Council, the National Institute of 10.13039/100005622Health Research, the 10.13039/100009250National Institute for Social Care and Health Research (Welsh Assembly Government), the 10.13039/501100000589Chief Scientist Office (10.13039/100012095Scottish Government Health Directorates), the 10.13039/100010269Wellcome Trust, (10.13039/501100000265MRC Grant No: MR/K006525/1). This work was supported by Health Data Research UK (grant ref:NIWA1), which is funded by the UK 10.13039/501100000265Medical Research Council, 10.13039/501100000266Engineering and Physical Sciences Research Council, 10.13039/501100000269Economic and Social Research Council, 10.13039/501100000272National Institute for Health Research (England), 10.13039/501100000589Chief Scientist Office of the Scottish Government Health and Social Care Directorates, 10.13039/501100010756Health and Social Care Research and Development Division (10.13039/100015846Welsh Government), 10.13039/501100001626Public Health Agency (Northern Ireland), 10.13039/501100000274British Heart Foundation (10.13039/501100000274BHF) and the 10.13039/100010269Wellcome Trust. This work was also supported by an 10.13039/501100000269ESRC award establishing the Administrative Data Research Centre Wales (ES/L007444/1). RF is supported by the 10.13039/100012068Health and Care Research Wales funded National Centre for Population Health and Wellbeing Research.

## Declaration of competing interest

The authors declare that they have no competing interests.

## Figures and Tables

**Figure:**
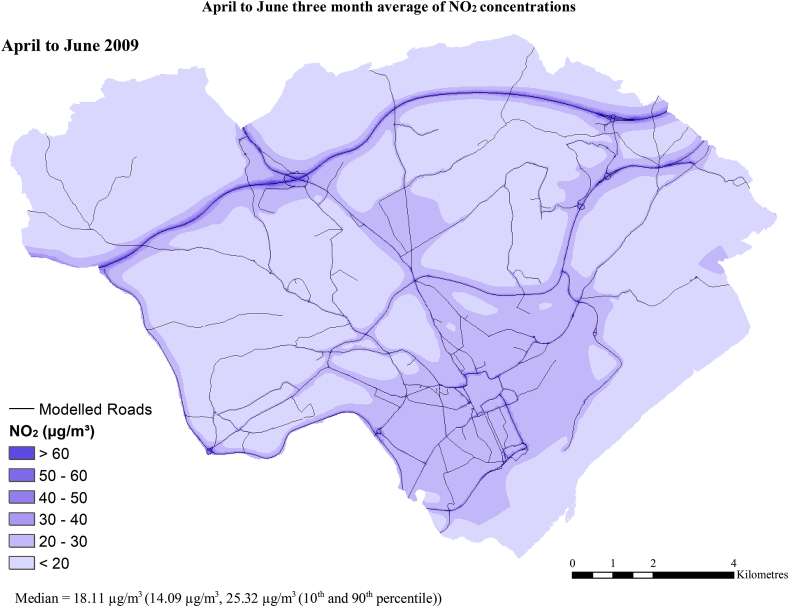


**Figure:**
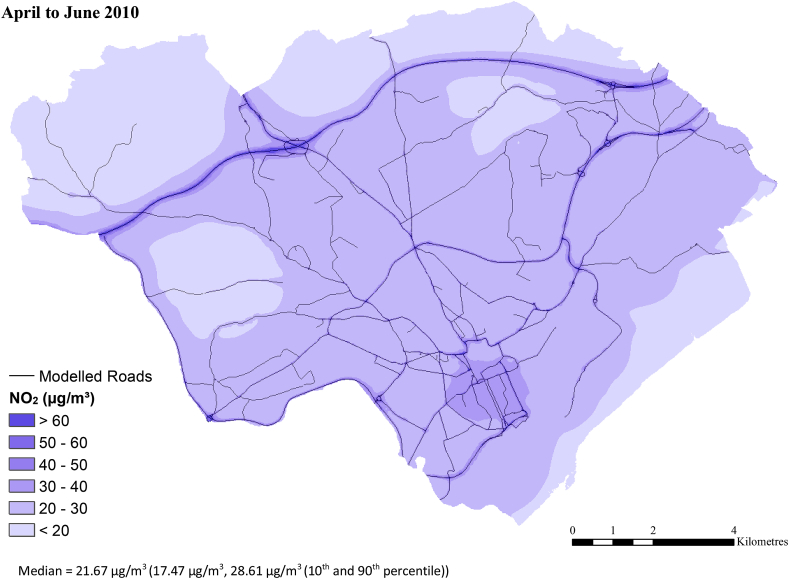


**Figure:**
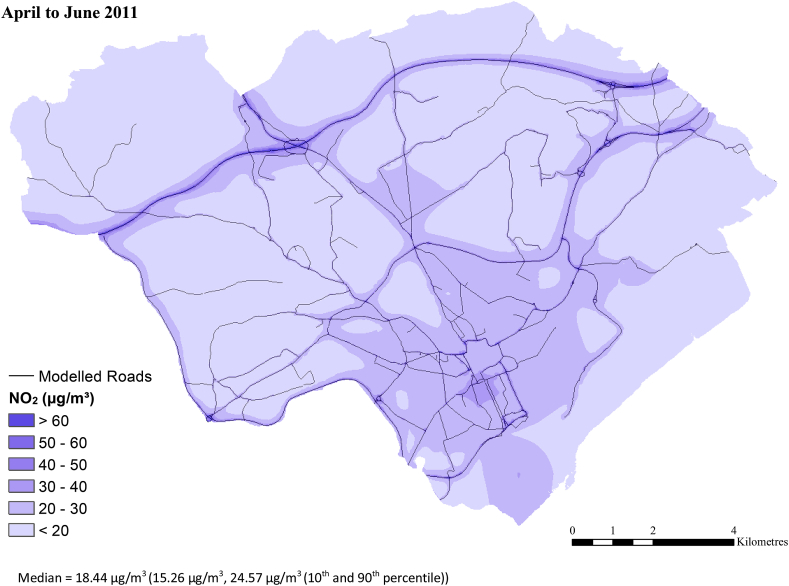


**Figure:**
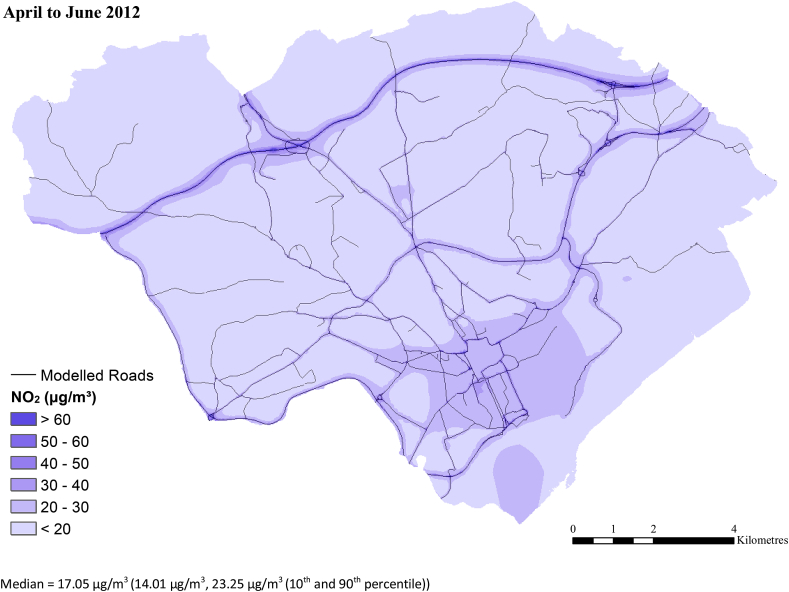


**Figure:**
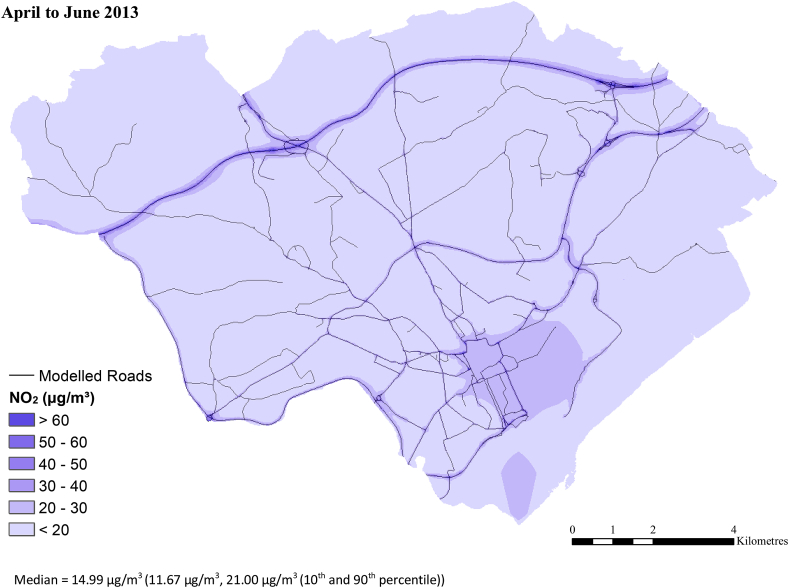


**Figure:**
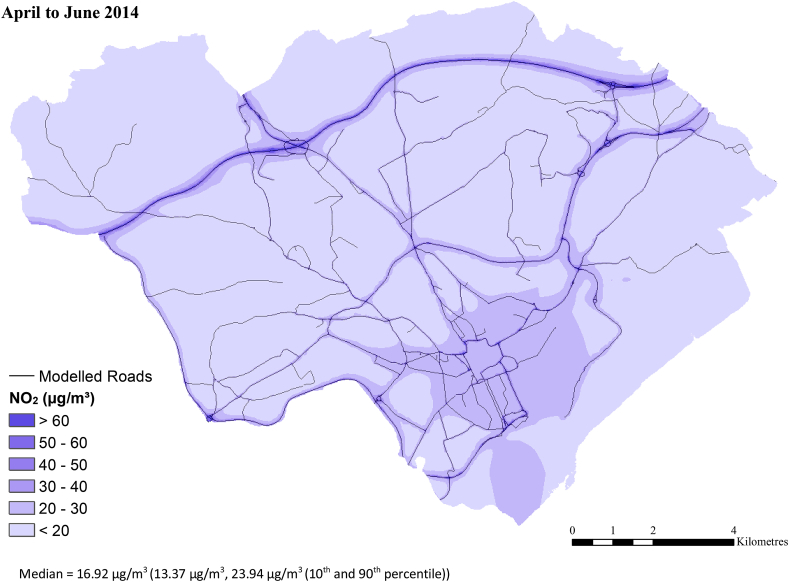


**Figure:**
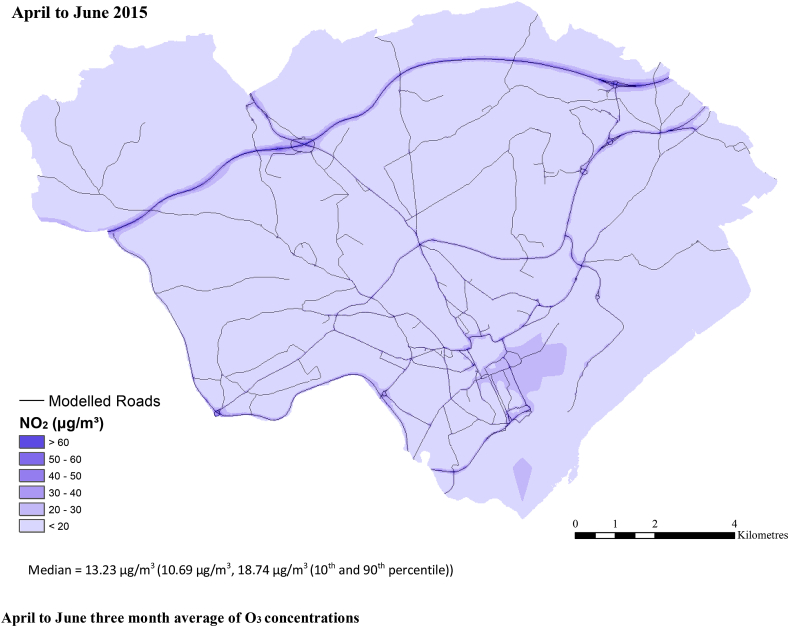


**Figure:**
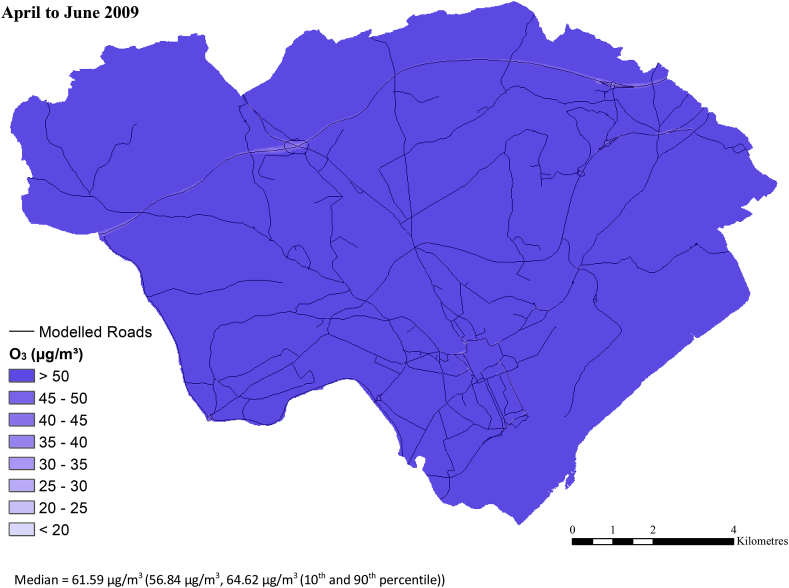


**Figure:**
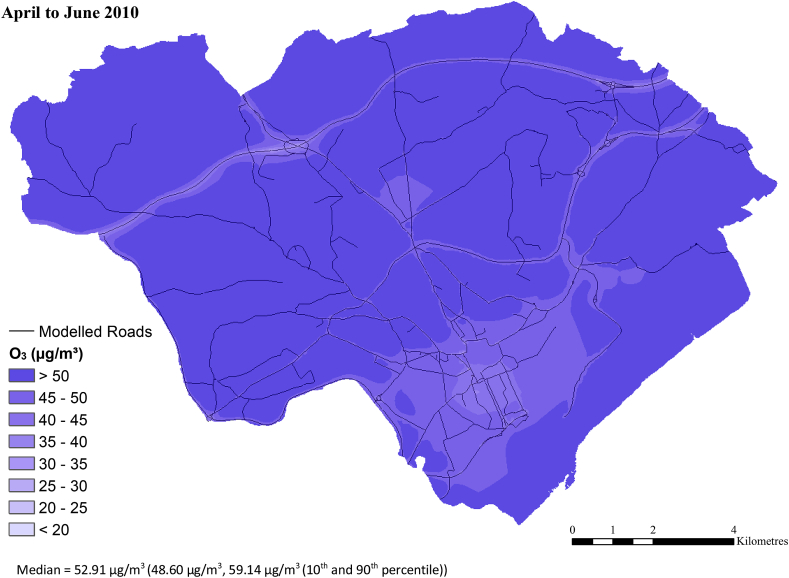


**Figure:**
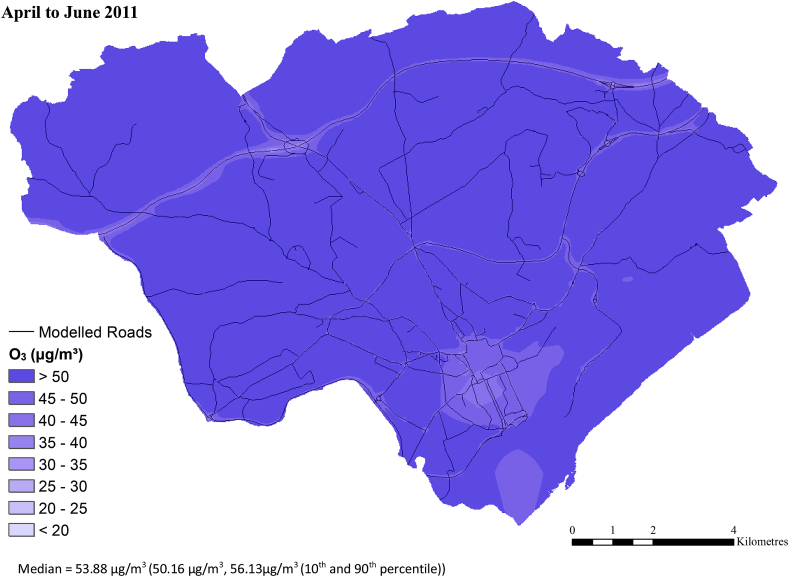


**Figure:**
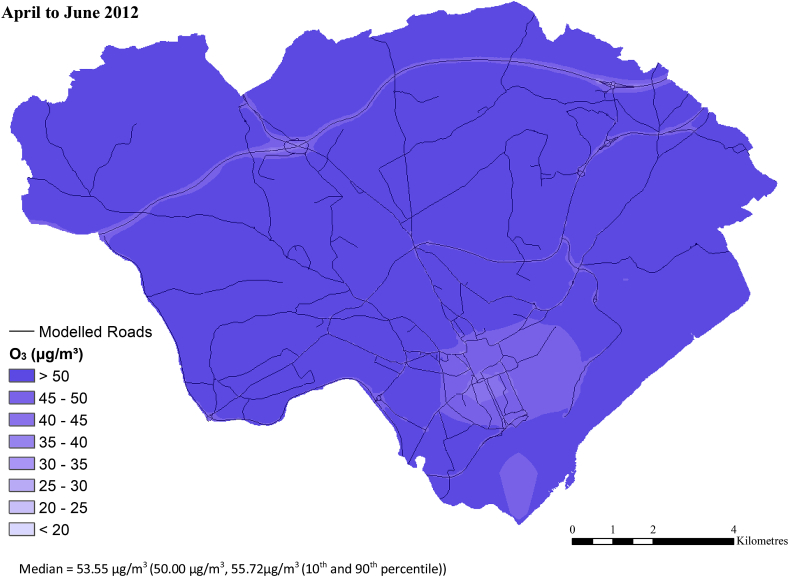


**Figure:**
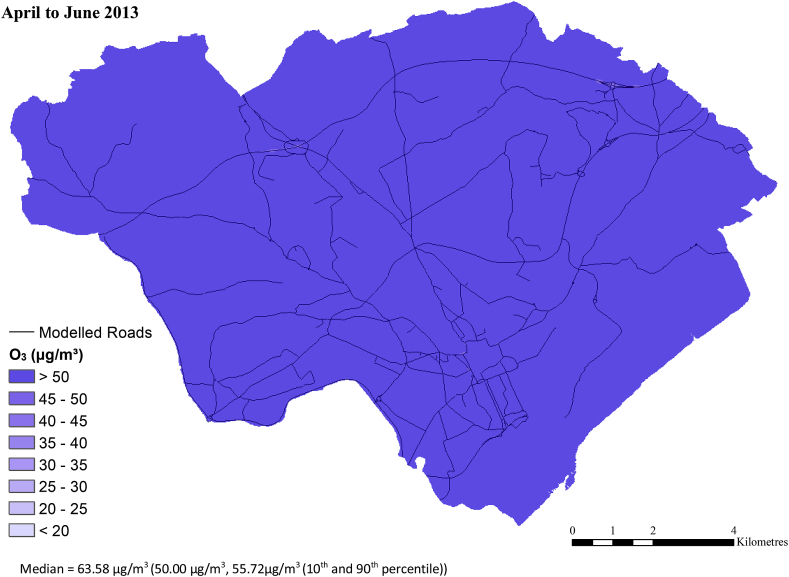


**Figure:**
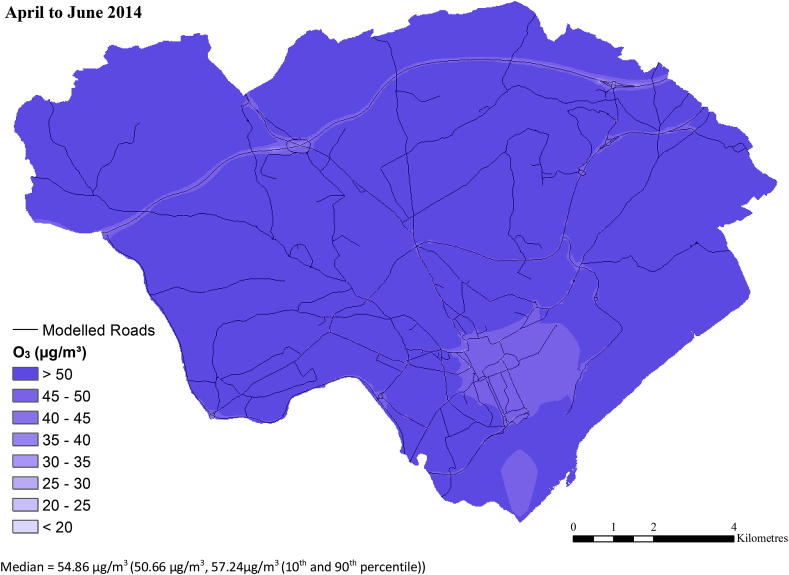


**Figure:**
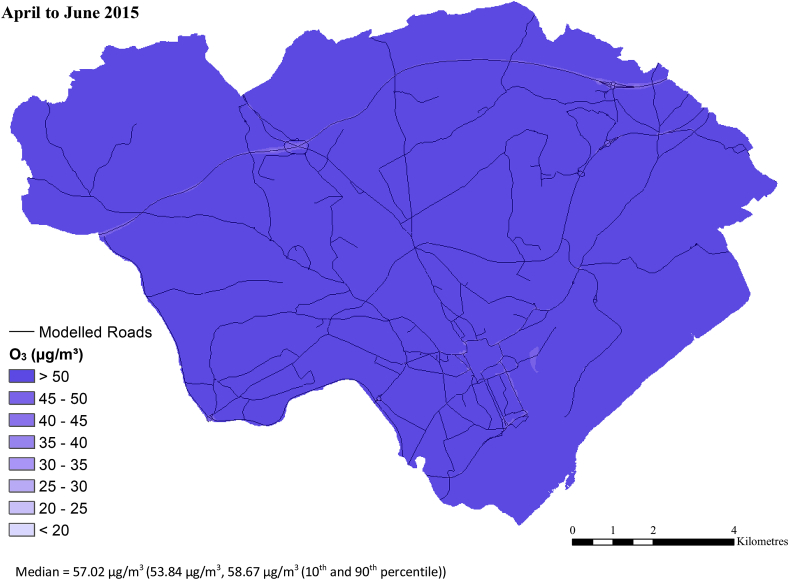


**Figure:**
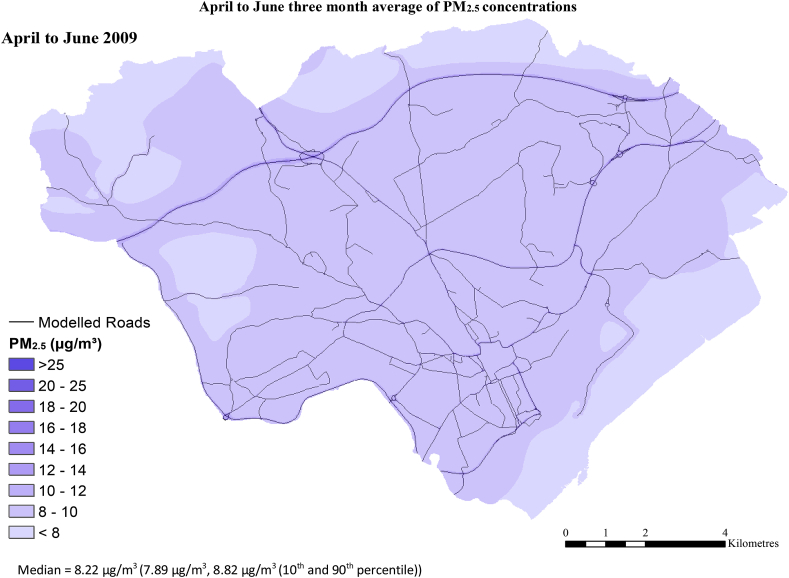


**Figure:**
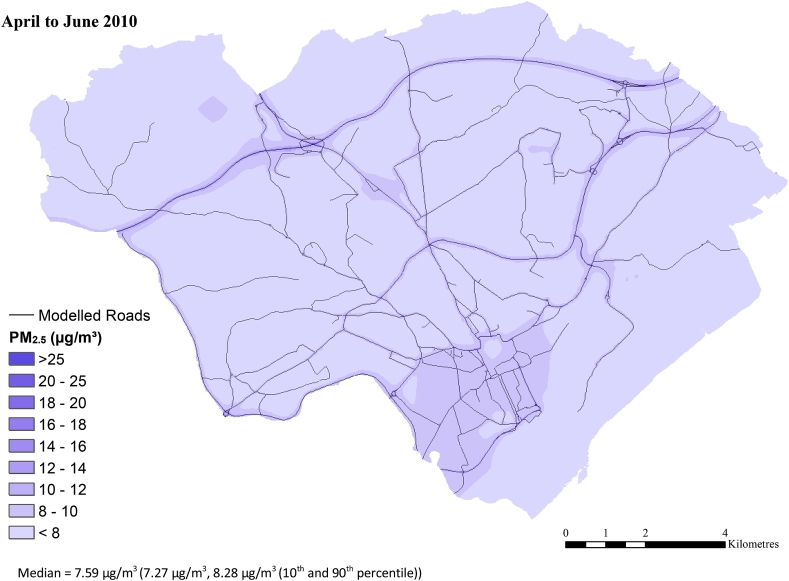


**Figure:**
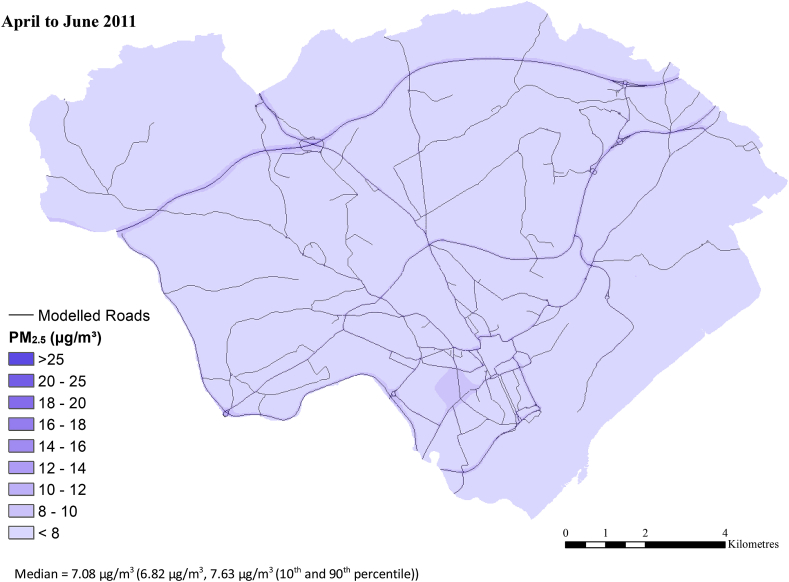


**Figure:**
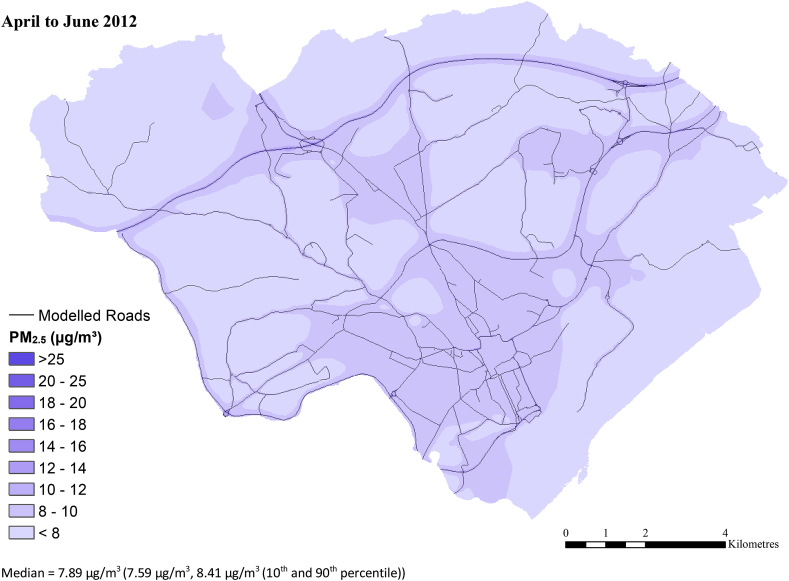


**Figure:**
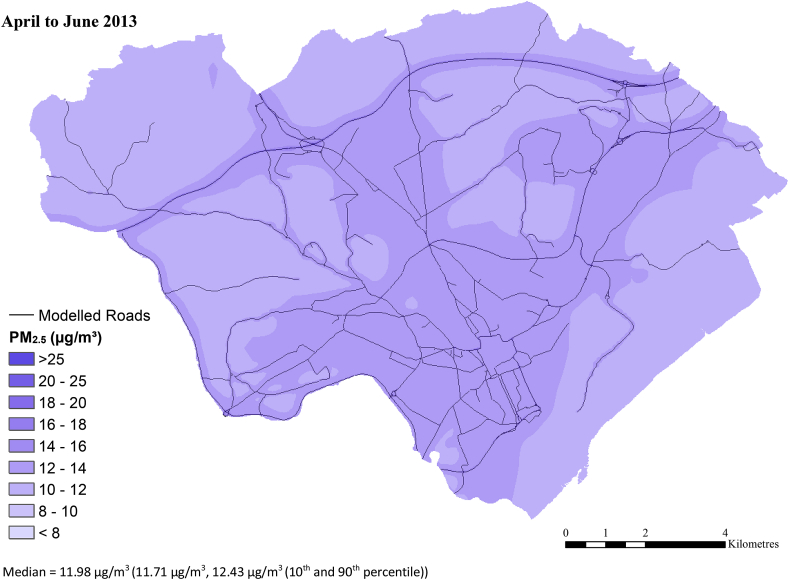


**Figure:**
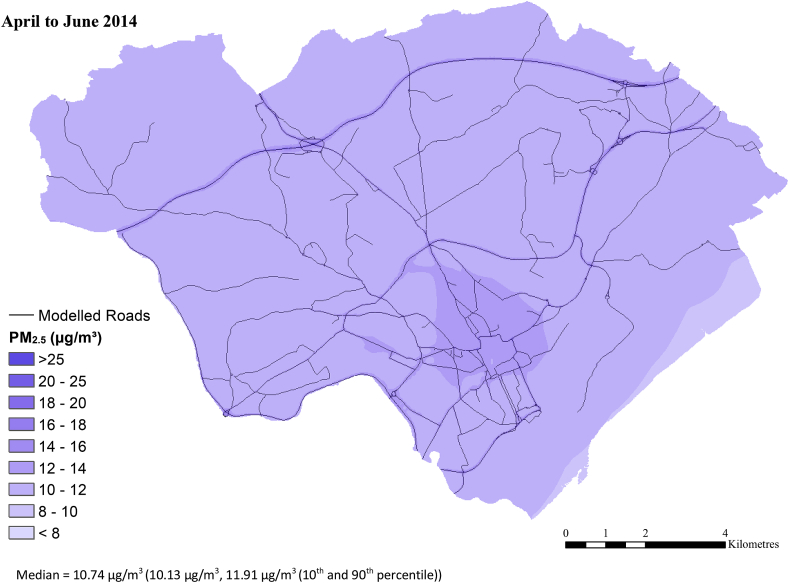


**Figure:**